# A comparative study of postmortem distribution and postmortem diffusion of tramadol in rabbits

**DOI:** 10.1038/s41598-022-25459-w

**Published:** 2023-01-30

**Authors:** Hongliang Su, Lingxiao Wang, Amin Wurita, Chao Zhang, Miaomiao Wu, Bin Li, Weifen Niu, Youmei Wang, Zhiwen Wei, Keming Yun

**Affiliations:** 1 School of Forensic Medicine, Shanxi Medical University, No. 56, Xinjian South Road, Yingze District, Taiyuan, 030001 China; 2 Key Laboratory of Forensic Toxicology, Ministry of Public Security, Beijing, 100192 China; 3 Department of Legal Medicine, College of Basic Medical Sciences, Inner Mongolia Medical University, Hohhot, 010010 China; 4 The People’s Procuratorate of Baoding, Baoding, 071000 China; 5 Department of Infection Management, School and Hospital of Stomatology, Shanxi Medical University, Taiyuan, 030001 China; 6 National Narcotics Laboratory, Drug Intelligence and Forensic Center, Ministry of Public Security, No. 18 Dongbeiwang West Road, Haidian District, Beijing, 100193 China

**Keywords:** Drug regulation, Toxicology

## Abstract

In recent years, the cases of tramadol intoxication have become more frequent in many countries. However, most of the previous studies have been based on cases of tramadol intoxication, and the detailed information on the differences between postmortem distribution and diffusion of tramadol remains unclear. To investigate this issue systematically, we established a postmortem distribution model and two postmortem diffusion models. Then, gas chromatography-mass spectrometry (GC/MS) was used to measure the concentrations of tramadol in various biological specimens of fluids and tissues. In postmortem distribution, the results showed an uneven distribution of tramadol in various biological specimens, and the concentrations of tramadol in urine were significantly higher than those in other fluids. In postmortem diffusion, the results showed a dosage-dependent increase of tramadol concentration in most specimens; at all time points from 0.25 to 6 h after postmortem administration, the concentrations of tramadol in fluids were not significantly different from those in tissues, and the concentrations of tramadol in urine were lower than those in both tissues and other fluids in most time points. We recommend a quantitative examination of the specimens of both fluids and tissues to provide more evidence for the forensic identification, and the realization that there is a correlation between the concentrations of fluids and tissues is important for determining antemortem and postmortem administration of tramadol. This information can serve as ancillary data in inferring the contribution of a drug to death in cases of suspected tramadol poisoning.

## Introduction

As a synthetic opioid, tramadol has been widely used for its pain-relieving effects^1–4^. It was originally manufactured in Germany in 1977^5,6^ and then in other countries some 20 years later. Tramadol, a pharmaceutical opioid, has been used as a painkiller for decades, but its non-medical use has caused a crisis of synthetic opioid overdoses in many subregions, in particular West, Central and North Africa, which requires urgent international attention^7^. Ojanperä et al. used the fatal toxicity index (FTI)—the absolute number of fatal poisonings caused by a particular drug—to evaluate the toxicity of 70 drugs and their relevance to fatal poisonings, and found that FTI of tramadol ranked 9th out of 70 drugs^8^. In a recent study in Denmark, the researchers analyzed opioid poisonings cases from 2006 to 2017 and found that 48 of the 408 deaths were caused by tramadol poisoning^9^.

Tramadol is used non-medically for the purposes of achieving pleasurable effects, i.e., to improve mood, prolonging the duration of sexual intercourse^10,11^, delaying the sensation of fatigue, and relieving pain, depression, anxiety, or other psychiatric disorders as a self-medication^7^. Therefore, there is a large market of tramadol for non-medical use, in which tramadol may be diverted from the licit production to the illicit markets, or be supplied by illicit manufactures at source. Due to its illicit and improper use, tramadol overdose-related intoxication and deaths are becoming frequent cases of drug poisoning in many countries^6,12–16^.

In cases of fatal intoxication, the interpretation of the probable cause of death relies mostly on the measurement of the concentrations of drugs. However, postmortem drug concentrations may vary according to the sampling site and the interval between death and postmortem specimen collection. This concept, known as postmortem redistribution (PMR), may complicate the interpretation of toxicological analyses. In fact, PMR may occur in either of the two cases: (1) antemortem (before death) administration of drug; (2) postmortem (after death) administration of drug^17–19^. Although PMR occurs in two cases likewise, the drug contributes to death differently, and the realization is important in forensic practice. Most previous research of PMR focuses on the cases of human unintentional death or suicide and falls into the category of antemortem administration of drug, so does our previous study of postmortem (antemortem administration of drug) redistribution of tramadol in poisoned rats^20^. Unlike conventional PMR, drug diffusion caused by postmortem administration has received little attention, i.e., drug was administered to dead individuals, which may occur when deaths from other reasons were disguised as acute tramadol intoxication. Thus, forensic scientists need to differentiate antemortem administration from postmortem administration for accurate identification.

To investigate this issue, we first explored the postmortem distribution of tramadol administered to live rabbits, simulating the state of individuals who died from acute intoxication. In order to determine whether postmortem diffusion occurs, and if so, the extent of passive diffusion, we designed two experiments to study the effects of dosage and length of time on postmortem diffusion by simulating the state of dead individuals to whom tramadol was administered. Finally, postmortem distribution and postmortem diffusion of tramadol were compared in rabbits, and the resulting correlation can be used to determine whether tramadol was administered before or after death.

## Materials and methods

### Drugs and chemicals

Standard tramadol (purity > 99.9%) was purchased from Institute of Forensic Science, Ministry of Public Security, China. Tramadol hydrochloride (50 mg/tablet) was purchased from Guangzhou Pui’s Pharmaceutical Co. Ltd, China. SKF_525A_, purchased from Standard Technology Development Co. Ltd, China, was prepared in methanol at a concentration of 1 g/L and used as the internal standard (IS). Both tramadol and IS were stored at 4 °C and protected from light. All other reagents were purchased from Sigma.

### Animal experiments

The animals used in this study were 30 male rabbits weighing 2.0 ± 0.3 kg, provided by the Laboratory Animal Center of Shanxi Medical University, China. All rabbits fasted for 24 h before the experiment. All animal experiments complied with the ARRIVE guidelines, were approved by Institutional Animal Care and Use Committee of Shanxi Medical University, and performed in accordance with the current relevant legislation in China.

In the experiment of postmortem distribution, we administered tenfold LD_50_ (LD_50_ = 228 mg/kg) of tramadol intragastrically (ig) to six rabbits to make sure that the rabbits die from an overdose of tramadol. The autopsy was carried out immediately after the rabbits’ death and the specimens were collected separately for the study of tramadol’s postmortem distribution.

In two experiments of postmortem diffusion, all 24 rabbits (drug-free) were euthanized with aeroembolism before administration of tramadol. 2 h after the animals’ death, we used 9 rabbits and 15 rabbits to study the effects of dosage and length of time on tramadol’s postmortem diffusion, respectively, simulating the death of individuals shortly before tramadol administration. In the dosage effect group, three dosages of 1/8 LD_50_, 1/4 LD_50_, and 1/2 LD_50_ of tramadol were ig administered to three subgroups (n = 3) respectively, and all rabbits were then placed at room temperature (20 °C). 0.25 h after postmortem administration, the autopsy was carried out and the specimens were collected separately for detection. In the time effect group, all 15 rabbits were given ig a 1/4 LD_50_ dosage of tramadol and placed at room temperature (20 °C). The autopsy was carried out at 5 different time points (0.25 h, 0.5 h, 1 h, 3 h, and 6 h, n = 3) after postmortem administration and the specimens were collected separately for detection.

### Extraction procedure

First, add 2 mL deionized water to each sample (1 mL or 1 g), homogenize the samples with a high-speed tissue homogenizer, and then add 20 μL (1 g/L) IS SKF_525A_ to the homogenate. Next, add 10% HCl to the homogenate samples (pH value of 1–2) to precipitate protein, and adjust pH value of the supernatant solutions to 10 by adding 10% NaOH. The analytes were extracted twice from the samples with diethyl ether. The resulting organic extract was concentrated to dryness under water bath of 40 °C and the residue was dissolved in 20 μL ethanol for GC/MS analysis. Some samples, such as stomach, were highly concentrated and had to be properly diluted before analysis.

### GC/MS

GC/MS analyses were performed on a Thermo Fisher Scientific TRACE DSQ system. Aliquots (1 μL) of the extract were injected into a DB-5 MS capillary column (30 m × 0.25 mm × 0.25 μm, J&W, Folsom, CA, USA). The adopted column temperature program was as follows: start with an initial temperature of 140 °C for 1 min, then rise to 250 °C at a rate of 30 °C/min, lastly, hold at 250 °C for 10 min. The carrier gas was helium with a constant flow at 1 mL/min. The temperature of ion source and transfer line was at 250 °C. Injection port temperature was also at 250 °C and the split injection mode was adopted (the split ratio was 10:1). The MS was operated in the selected ion monitoring mode, and the following ions were monitored: quantifier ion *m/z* 58 and qualifier ions *m/z* 77 and *m/z* 263 for tramadol; quantifier ion *m/z* 86 and qualifier ions *m/z* 99 and *m/z* 353 for the IS.

### Method validation

Method validation was performed according to the guidelines of bioanalytical method validation introduced by U.S. FDA^21^. In the present study, to validate the results of bioanalytical method and facilitate the subsequent comparative analysis, we added standard tramadol and IS to each type of blank matrix samples of rabbits.

The linearity of the calibrator responses was estimated based on linear regression analysis, ranging from 0.05 to 96 μg/mL or μg/g, with a linearity expected to be > 0.99. Limit of detection (LOD) and lower limit of quantitation (LLOQ) were determined to estimate the sensitivity of the analytical method and calculated as the concentration of the inject sample to yield a signal-to-noise ratio of 3 and 10, respectively. For both LOD and LLOQ, the retention time was required to be within ± 2% and the ion ratio within ± 20% of the determined value of higher concentration standard samples, for valid results to be obtained.

Accuracy and precision were evaluated at three quality control (QC) concentrations (1, 16, and 64 μg/mL or μg/g) in six replicates (n = 6, respectively). Before each assay, we prepared QC samples were prepared before each assay by spiking blank samples with the tramadol reference standard solution at the determined concentrations. Accuracy was represented by the relative error of the mean (REM, in %), which was expected to lie within ± 15%. Intra- and inter-day precisions were represented by relative standard deviations (RSD, in %), which were supposed to be lower than 15%.

We compared the analytes with six concentrations of 0.5, 1, 4, 20, 40, and 80 μg/mL or μg/g to determine the recovery rates of tramadol from each matrix during the extraction process. And the analytes at each concentration were prepared in six replicates (n = 6, respectively).

### Statistical analysis

Statistical analyses were performed with the statistical program of SPSS 11.5 and the data was represented by mean ± standard deviation (SD). As with all statistical measures, a *P* value of < 0.05 was considered significant in this study.

### Ethics approval

All experiments were approved by Institutional Animal Care and Use Committee of Shanxi Medical University, and performed in accordance with the current relevant legislation in China. The authors complied with the ARRIVE guidelines.

## Results

### Method validation

A calibration curve for each biological matrix was generated. The calibration curves of tramadol were linear with a linearity > 0.99. The corresponding LOD and LLOQ of each biological matrix were gained, reaching at least 0.05 μg/mL (or μg/g) and 0.1 μg/mL (or μg/g), respectively. Accuracy and precision were determined based on the QC data. In our study, both % REM and the intra- and inter-day % RSD were found to be lower than 15%. At concentrations of 0.5, 1, 4, 20, 40, and 80 μg/mL (or μg/g), the ranges of average extraction recoveries of tramadol in all samples were: (92.2 ± 5.2)%–(109.4 ± 8.6)%, (96.7 ± 3.1)%–(107.6 ± 5.1)%, (98.0 ± 2.4)%–(104.6 ± 6.0)%, (94.8 ± 7.0)%–(105.8 ± 9.1)%, (95.5 ± 5.7)%–(102.7 ± 7.8)%, and (93.4 ± 5.9)%–(105.3 ± 6.3)% respectively. In conclusion, all validation results of GC/MS have met the requirements of bioanalytical method.

### Postmortem distribution of tramadol in rabbits dying of poisoning

**Figure 1:**
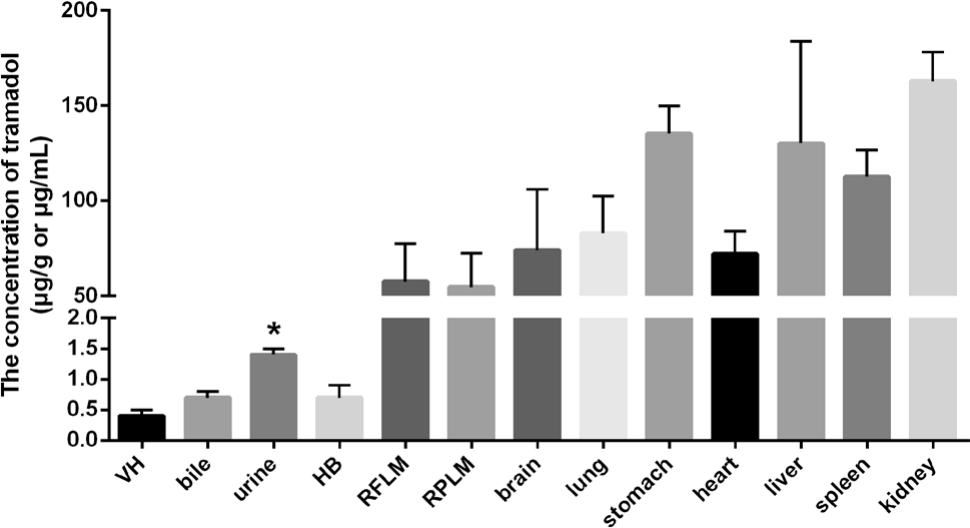
Postmortem distribution of tramadol in rabbits dying of poisoning. *VH* vitreous humor, *HB* heart blood, *RFLM* right forelimb muscles, *RPLM* right posterior limb muscles. Data was represented by mean ± standard deviation. *P < 0.05, compared with the concentrations of tramadol in other fluids.

The results of postmortem distribution showed that tramadol was unevenly distributed in various biological specimens (Fig. 1). The concentrations of tramadol detected in the specimens of rabbits dying of poisoning were as follows: 163.0 ± 15.3 μg/g in kidney, 135.3 ± 14.5 μg/g in stomach, 130.2 ± 53.7 μg/g in liver, 112.5 ± 14.2 μg/g in spleen, 83.0 ± 19.4 μg/g in lung, 73.9 ± 32.2 μg/g in brain, 71.9 ± 12.2 μg/g in heart, 57.6 ± 19.9 μg/g in right forelimb muscles, 54.8 ± 17.4 μg/g in right posterior limb muscles, 1.4 ± 0.1 μg/mL in urine, 0.7 ± 0.1 μg/mL in bile, 0.7 ± 0.2 μg/mL in heart blood, and 0.4 ± 0.1 μg/mL in vitreous humor (Fig. 1).

It is noteworthy that the concentrations of tramadol in tissues (54.8–163.0 μg/g) were significantly higher than those in fluids (0.4–1.4 μg/mL). Additionally, the concentrations of tramadol in urine (1.4 μg/mL) were significantly higher than those in other fluids (0.4–0.7 μg/mL). These results suggested that, apart from conventional markers of blood, liver, kidney, and brain, urine also has the potential for identifying the individuals who died of poisoning, i.e., antemortem administration.

### Tramadol’s postmortem diffusion in rabbits

24 rabbits (drug-free) were euthanized with aeroembolism. 2 h later, 9 rabbits and 15 rabbits were used to study the effects of dose and length of time on tramadol’s postmortem diffusion, respectively. Postmortem diffusion of tramadol was confirmed in both experiments.

### The effect of dosage on tramadol’s postmortem diffusion in rabbits

3 subgroups were given a 1/8 LD_50_, 1/4 LD_50_, and 1/2 LD_50_ dosage of tramadol (n = 3) by ig respectively, and placed at room temperature (20 °C). 0.25 h after the postmortem administration, the autopsy was carried out and the specimens were collected separately for detection.

**Figure 2:**
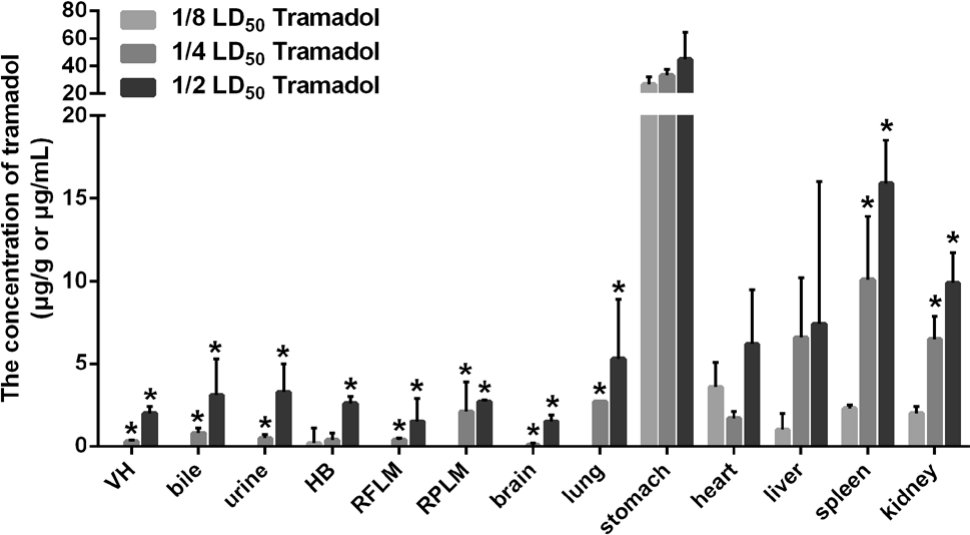
The effect of dosage on postmortem diffusion of tramadol in rabbits. *VH* vitreous humor, *HB* heart blood, *RFLM* right forelimb muscles, *RPLM* right posterior limb muscles. Data was represented by mean ± standard deviation. *P < 0.05, compared with the concentrations of tramadol in the dosage of 1/8 LD_50_.

Tramadol was detected in all biological specimens at most dosages except that the concentrations of tramadol in vitreous humor, bile, urine, right forelimb muscles, right posterior limb muscles, brain, and lung were lower than LLOQ at the dosage of 1/8 LD_50_ (Fig. 2). A dosage-dependent effect was confirmed, because in the same kind of specimens, the higher the dosage of postmortem administration was, the higher the concentrations of tramadol were (^*^P < 0.05, compared with the concentrations of tramadol at the dose of 1/8 LD_50_, Fig. 2). Although there was no statistically significant difference in tramadol’s concentrations in stomach, heart, and liver at three different dosages, an increasing trend was observed. These results suggested that increasing the dosage in postmortem administration can promote the postmortem diffusion of the drug.

### The effect of length of time on tramadol’s postmortem diffusion in rabbits

15 rabbits were given a 1/4 LD_50_ dosage of tramadol by ig respectively, and placed at room temperature (20 °C). The autopsy was carried out at five different time points (0.25 h, 0.5 h, 1 h, 3 h, and 6 h, n = 3) after postmortem administration and the specimens were collected separately for detection.

**Figure 3:**
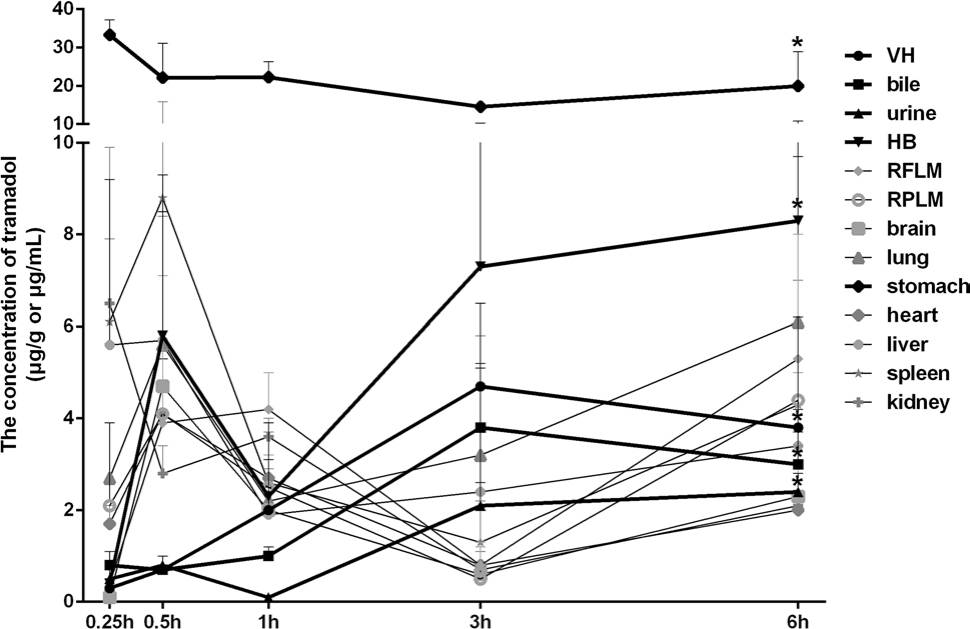
The effect of length of time on postmortem diffusion of tramadol in rabbits. *VH* vitreous humor, *HB* heart blood, *RFLM* right forelimb muscles, *RPLM* right posterior limb muscles. Data was represented by mean ± standard deviation. *P < 0.05, compared with the concentrations of tramadol at 0.25 h.

Tramadol was detected in all biological specimens during 0.25–6 h after the postmortem administration (Fig. 3). It was noteworthy that the concentrations of tramadol in fluids (including vitreous humor, bile, urine, and heart blood) obviously increased at 6 h after the postmortem administration (^*^P < 0.05, compared with the concentrations of tramadol at 0.25 h, Fig. 3). In contrast, the concentrations of tramadol in stomach decreased (^*^P < 0.05, compared with the concentrations of tramadol at 0.25 h, Fig. 3). As to other tissues (kidney, liver, spleen, lung, brain, heart, right forelimb muscles, right posterior limb muscles), there was no statistically significant difference in concentrations at different time points from 0.25 to 6 h (Fig. 3). These results indicated that fluids may be more sensitive than tissues in assessing postmortem diffusion of drug.

Additionally, the concentrations of tramadol in fluids (0.1–8.3 μg/mL) were not significantly different from those in tissues (0.1–8.8 μg/g, except stomach). Despite the general upward trend, the concentrations of tramadol in urine were lower than those in both tissues and other fluids at most time points during 0.25 h to 6 h after postmortem administration.

## Discussion

IN medicolegal investigations, the reference values (i.e., therapeutic, toxic, or lethal concentrations) are often used by forensic pathologists and toxicologists to assess the contribution of a drug to the death in fatalities. Therefore, the measurement of tramadol’s concentration is important for identifying the cause of death in the postmortem investigation. It was reported that the therapeutic concentration of tramadol in blood ranged from 0.1 to 0.3 μg/mL approximately^1,13,22^. If the concentration exceeds 1.0 μg/mL, the chances of toxic effects will increase^23–25^. In the present study, tramadol was widely distributed in all organs after ig administration (Fig. 1). With a mean of 0.7 μg/mL, the heart-blood concentration of tramadol seems not high. A probable explanation was that the relatively high dosage (10 LD_50_) led to rapid death in individuals; as a result, the drug had not been fully absorbed and sufficiently distributed in blood at the time of individuals’ death. This was confirmed by our previous study of postmortem redistribution^20^, in which the concentrations of tramadol were higher in blood (7.0 μg/mL) instead, though a lower dose (1/2 LD_50_) was used, possibly because of a longer survival time. Previous studies also showed that the observed concentration of a neuro-psychotropic drug in plasma may not accurately reflect the pharmacologic effects^26–28^. A recent study of postmortem drug distribution indicated that the detected concentrations of fifteen synthetic opioids, with the exception of norfentanyl, were higher in brain tissue than in blood^29^. In the present study, the concentration of tramadol in brain reached a high of 73.9 μg/mL, although it was 0.7 μg/mL in blood (Fig. 1). Additionally, the concentration of tramadol (1.4 μg/mL) in the specimen of urine was significantly higher than those in other fluids (0.4–0.7 μg/mL), which was consistent with previous cases of fatal intoxication due to tramadol alone^25,30^, indicating that the specimen of urine, like the conventional markers of blood, liver, kidney, and brain, can also be a sensitive marker to assess the individuals who died of poisoning, i.e., antemortem administration. These results suggested that quantitative analysis of different specimens is necessary for toxicological identification, because various specimens can provide more proof of drug intoxication in lethal cases. Moreover, it is important for medical toxicologists handling drug-related cases to note that postmortem administration of drugs (such as postmortem intravenous infusion or ig administration) may also lead to a lethal blood concentration of drugs, while the concentrations in tissues remain low. In such cases, an overdose of drugs was not the cause of death, although it seemed so, because postmortem diffusion may also lead to a lethal blood concentration. Thus, it is unreliable to assess the contribution of a drug to individuals’ death through its concentration in blood only, because it is also necessary to identify the correlation between the concentration of fluids and that of tissues before the cause of death can be determined, otherwise erroneous conclusions may be drawn.

PMR generally refers to the changes of drug concentrations occurring after death^19^, and involves the redistribution of drugs caused by concentration gradients, typically from high to low concentrations^31,32^. PMR may be influenced by two factors: (1) diffusion through blood vessels towards the surroundings and/or transparietal diffusion from organs to blood vessels; (2) spontaneous degradation and/or microorganism decomposition^32,33^. The extent of PMR will be influenced by factors of pharmacological characteristics (such as absorption route, state of ionization, volume of distribution, protein binding affinity, and lipophilicity), postmortem interval (PMI), site of drug sampling, and storage temperature of the corpse^19,32,34^. As our previous study demonstrated, both PMI and storage temperature will affect the PMR of tramadol^20^. In fact, PMR may occur in either of the two cases^17–19^: (1) antemortem administration of drug; (2) postmortem administration of drug. Although PMR occurs in two cases likewise, the drug contributes to death differently, and the realization is important in forensic practice. Human unintentional death or suicidal cases fall into the category of antemortem administration of drug, in which a rather large amount of drug is given perorally shortly before death. Drug will be rapidly absorbed and then widely distributed into well-perfused tissues via the circulatory system, such as liver, kidney, spleen, and lung. Once individuals die, a drug reservoir will be formed in the stomach and/or gut as a result of incomplete absorption of drug at the time of death.

Unlike most previous studies, this study focused on postmortem administration of drug, which was administered to dead individuals, and we investigated whether postmortem diffusion occurred as well as the extent of postmortem passive diffusion. Since tramadol was administered by ig to dead rabbits, the only drug absorption and distribution took place postmortem in the stomach. Therefore, a considerable “reservoir” of the substance was formed in the stomach, providing a concentration gradient for passive diffusion. Our results revealed that: (1) tramadol concentration showed a dosage-dependent increase in most specimens (Fig. 2); (2) tramadol concentration detected in stomach decreased over time, and increased in fluids (including vitreous humor, bile, urine, and heart blood); no significant difference was detected in other tissues (including right forelimb muscles, right posterior limb muscles, brain, lung, stomach, heart, liver, spleen, and kidney) during 0.25 h to 6 h after postmortem administration (Fig. 3). Tramadol was more readily redistributed in fluids possibly because the drug was dissolved^19,35^, which is consistent with previous studies in which blood, as a matrix on PMR, was sensitive to the change of drug concentration^16,36^. Additionally, it is noteworthy that the concentrations of tramadol in urine were lower than those in both tissues and other fluids at most time points during 0.25 h to 6 h after postmortem administration. The above-mentioned results indicated that postmortem ig administration of tramadol can likewise lead to considerable drug PMR (postmortem diffusion) in the body, which should be taken into account in forensic practice to avoid false conclusions.

Hence, it is necessary that the specimens of both fluids and tissues be quantitatively examined to provide more evidence for forensic identification, and the realization that there is a correlation between the concentrations of fluids and tissues is important for differentiating antemortem administration from postmortem administration. When administered by ig to live animals, drug was rapidly absorbed and then distributed into well-perfused tissues via the circulatory system due to the high Vd and liposolubility^37,38^; therefore, concentrations were high in fluids (especially urine and blood) and tissues (especially brain, liver, and kidney), and the differences in the concentrations of tramadol between fluids and tissues were significant. When administered by ig to dead animals, drug accumulated in the stomach alone and diffused through gastric wall towards the surrounding organs and blood vessels, so the differences in the concentrations of tramadol between fluids and tissues were not significant (though fluids are supposed to be more sensitive than tissues in assessing postmortem diffusion of drug), and the concentrations of drug in urine were lower than those in other fluids and tissues at most time points. This information can serve as ancillary data in inferring the contribution of a drug to death in medicolegal investigations.

## Conclusion

The present study systematically explored the postmortem distribution and postmortem diffusion of tramadol in rabbits, aiming to differentiate antemortem administration from postmortem administration of tramadol through comparing tramadol concentrations in fluids and tissues. Our study provides valuable information for forensic toxicologists in assessing cases of suspected tramadol poisoning.

## Data Availability

All data generated during the study appear in the submitted article.
